# Decrease in the expression of muscle-specific miRNAs, miR-133a and miR-1, in myoblasts with replicative senescence

**DOI:** 10.1371/journal.pone.0280527

**Published:** 2023-01-17

**Authors:** Kaori Shintani-Ishida, Riko Tsurumi, Hiroshi Ikegaya

**Affiliations:** Department of Forensic Medicine, Graduate School of Medical Science, Kyoto Prefectural University of Medicine, Kyoto, Japan; Northwest University, UNITED STATES

## Abstract

Muscles that are injured or atrophied by aging undergo myogenic regeneration. Although myoblasts play a pivotal role in myogenic regeneration, their function is impaired with aging. MicroRNAs (miRNAs) are also involved in myogenic regeneration. MiRNA (miR)-1 and miR-133a are muscle-specific miRNAs that control the proliferation and differentiation of myoblasts. In this study, we determined whether miR-1 and miR-133a expression in myoblasts is altered with cellular senescence and involved in senescence-impaired myogenic differentiation. C2C12 murine skeletal myoblasts were converted to a replicative senescent state by culturing to a high passage number. Although miR-1 and miR-133a expression was largely induced during myogenic differentiation, expression was suppressed in cells at high passage numbers (passage 10 and/or passage 20). Although the senescent myoblasts exhibited a deterioration of myogenic differentiation, transfection of miR-1 or miR-133a into myoblasts ameliorated cell fusion. Treatment with the glutaminase 1 inhibitor, BPTES, removed senescent cells from C2C12 myoblasts with a high passage number, whereas myotube formation and miR-133a expression was increased. In addition, primary cultured myoblasts prepared from aged C57BL/6J male mice (20 months old) exhibited a decrease in miR-1 and miR-133a levels compared with younger mice (3 months old). The results suggest that replicative senescence suppresses muscle-specific miRNA expression in myoblasts, which contributes to the senescence-related dysfunction of myogenic regeneration.

## Introduction

The regeneration of skeletal muscle involves repairing muscles that have been injured or atrophied by aging or disease. Muscle regeneration following myotrauma is triggered by the activation of resident muscle satellite (stem) cells (MuSCs). Activated MuSCs proliferate as myoblasts and induce the expression of muscle-specific genes, including myoblast determination protein 1 (MyoD1) [1], myogenin [2], and myosin heavy chain (MHC) [3], to promote myocyte differentiation. The myocytes migrate and fuse with other myocytes to form multinucleated myotubes that mature into myofibers or with damaged myofibers. The regenerative capacity of skeletal muscle declines with advancing age, and the impaired function of senescent MuSCs is partly responsible [4–6].

MicroRNAs (miRNAs) are short (~22 n) non-coding RNAs that post-transcriptionally regulate gene expression by binding to the 3′ untranslated region of target mRNAs [7, 8]. Over 1000 miRNAs are present in the human genome, and many exhibit tissue- or developmental stage-specific expression patterns [9, 10]. Muscle-specific or muscle-enriched miRNAs include miRNA (miR)-1, miR-133a, miR-133b, miR-206, miR-208a, miR-208b, miR-486, and miR-499 [11]. MiR-1, miR-133a/b, and miR-206 control the proliferation and differentiation of myoblasts [12]. MiR-1 accelerates myoblast differentiation by reducing the expression of histone deacetylase 4 (HDAC4), which inhibits MyoD1 [13]. MiR-206, which also targets HDAC4 [14], is skeletal muscle-specific, while miR-1 is both skeletal and cardiac muscle-specific. MiR-133a/b promotes myoblast proliferation by downregulating the serum response factor (SRF) [15]. MiR-1/miR-133a or miR-206/miR-133b is co-transcribed in the bicistronic transcript [15]. The MiR-206/133b cluster is likely to be dispensable for skeletal muscle regeneration because of the overlapping functions of the related miR-1/133a clusters [16]. MiR-208b and miR-499 are intronic miRNAs encoded in the myosin genes *Myh7* and *Myh7B*, respectively, and they control muscle gene expression and performance [17]. Although miR-486 is expressed in other tissues as well (muscle-enriched miRNA), it is upregulated during myoblast differentiation and downregulates Pax7 as well as miR-1/miR-206 [18]. MiR-208a is not expressed in skeletal muscles (cardiac muscle-specific expression).

There are several reports that miR-1 and miR-133a, among these muscle-specific/enriched miRNAs, vary during aging. In the right atrial appendage samples from patients undergoing coronary artery bypass grafting, miR-1 and miR-133 expression levels were lower in aged patients (≥65 years old) than in adult patients (<65 years old) [19]. Quantitative analysis of miRNAs from the tibialis anterior (TA) muscle of adult mice (6 months old) and aged mice (24 months old) revealed that several miRNAs, including miR-133a, decreased with aging [20]. However, miR-1 and miR-133a alterations in MuSCs during the aging process have not been reported.

In this study, we determined whether miR-1 and miR-133a expression in MuSCs is altered with aging and examined the mechanism through which altered miRNA expression contributes to aging-impaired regeneration; C2C12 murine skeletal myoblasts with a high passage number were used as an aging model [21].

## Materials and methods

### Cell culture and passage

Mouse C2C12 cells were provided by the RIKEN BioResource Research Center through the National BioResource Project of the MEXT/AMED (Ibaraki, Japan). The cells were cultured in the growth medium (GM) consisting of Dulbecco’s modified Eagle medium (DMEM) (Invitrogen, Life technologies Japan, Tokyo, Japan) supplemented with 10% fetal bovine serum substitute EquaFETAL (Atlas Biologicals, Fort Collins, CO, USA), at 37°C and 5% CO_2_ until the cells reached 60%–70% confluency. For each passage, the cells were trypsinized, diluted ten times, and seeded into new dishes for 72 h. Cells from the second passage were stored in a serum-free cell preservation medium Bambanker® (GC Lymphotec, Tokyo, Japan) at −80°C. For the experiments, the stock cells were thawed (passage 0) and passaging was repeated until a high passage number (passage 10 or passage 20) was achieved. Myogenic differentiation was induced by changing the GM to a differentiation medium (DM) consisting of 2% horse serum (Invitrogen^TM^) with DMEM. The DM was changed every other day. After 7 days, myotube formation was evaluated.

### Transfection

A 5 μM miRNA mimic, synthesized for miR-1 or miR-133a (AccuTarget^TM^ Human miRNA mimic, Bioneer Daejeon, Korea), was transfected into cells using Lipofectamine RNAiMAX Transfection Reagent (Invitrogen) following the manufacturer’s instructions. AccuTarget^TM^ miRNA mimic Negative Control #1 (Bioneer) was used as control miRNA.

### Senolysis

Cells were seeded into 60-mm culture dishes and 96-well black plates. Cells at 60%–70% confluence were treated with GM containing 10 μM BPTES [Bis-2-(5-phenylacetamido-1,3,4-thiadiazol-2-yl)ethyl sulfide] (AdipGen, Füellinsdorf、Switzerland) for 48 h. The 96-well plates were used to determine the number of viable cells; the dishes were used for miRNA quantitation. Cells on the other dishes were re-seeded into new 60-mm culture dishes at 5 × 10^5^ and grown to 60%–70% confluence and differentiation was induced by DM exchange. The cells were collected for Western blot analysis and immunostaining with antibodies against MHC after incubation with DM for 7 days.

### Immunostaining

The cells were fixed by incubation in cold methanol for 2 min and washed three times with phosphate-buffered saline (PBS). They were subsequently incubated with PBS containing 1% bovine serum albumin (BSA) (Sigma-Aldrich, St. Louis, MO, USA) at room temperature for 30 min followed by incubation with an anti-MHC monoclonal antibody (Clone MF20, R&D Systems, Minneapolis, MN, USA) at 4°C overnight. The antibody was diluted 1000-fold with PBS containing 1% BSA. The cells were washed and then incubated with Alexa Fluor 568–conjugated secondary antibody (ab175701, abcam, Cambridge, UK) at room temperature for 1 hour. After washing, they were mounted with a medium containing 4′,6-Diamidino-2-phenylindole dihydrochloride (DAPI) (Vector Laboratories, Burlingame, CA, USA). Using a fluorescence microscope (IX73, Olympus, Tokyo, Japan), four images were randomly selected from each well. The nuclei were counted using imaging software (cellSens, Olympus). The percentage of nuclei in the myotubes with MHC-positive staining was calculated as the fusion index.

### Quantitative reverse transcription (RT)-PCR

The relative quantities of miR-1 and miR-133 were determined using quantitative RT-PCR. Total RNA was extracted from cells using ISOGEN extraction reagent (Nippon Gene, Tokyo, Japan). Quantitative RT-PCR for miR-1 and miR-133a was performed using a TaqMan™ MicroRNA Assay kit (assay ID: 002222 for hsa-miR-1, 002246 for hsa-miR-133a) according to the manufacturer’s protocol. U6 snRNA was used as an endogenous control (assay ID: 001973). The threshold cycle (CT) values for the genes were determined with a StepOnePlus™ Real-Time PCR System (Thermo Fisher Scientific, Tokyo, Japan). The amount of the target gene relative to the endogenous control gene was normalized with each control sample by the ΔΔCT method.

### Western blot analysis

Cells were sonicated in an ice-cold solution of 320 mM sucrose, 10 mM Tris–HCl (pH 7.4), 1 mM EDTA, 50 mM NaF, 1 mM Na_3_VO_4_, and a protease inhibitor cocktail (P8340, Sigma-Aldrich). The lysates were solubilized in Laemmli sample buffer and subjected to 10% and 15% SDS-PAGE for MHC, MyoD, Myogenin, and β-tubulin and for p16^INK4a^ and p21, respectively. After transfer, the membranes were incubated with antibodies specific to MHC (Clone MF20, R&D Systems), MyoD (18943-1-AP, Proteintech, Tokyo, Japan), β-tubulin (ab179513, abcam), p16^INK4a^ (10883-1-ap, Proteintech), or p21 (28248-1-AP, Proteintech). The primary antibodies were diluted 1000-fold with 1% BSA in Tris-buffered saline containing Tween-20 (BSA/TBS-T). The secondary antibody, horseradish peroxidase-conjugated goat anti-mouse or anti-rabbit IgG (Promega, Madison, WI), was diluted 5000-fold with 1% BSA/TBS-T. The immune complexes were detected with chemiluminescent reagents (Western Lightning-ECL; PerkinElmer, Waltham, MA). The band intensities were measured using the ChemiDoc Touch imaging system (BioRad Laboratories, Hercules, CA, USA).

### Plate assay

SPiDER-βGal cellular senescence plate assay kit (Dojindo Laboratories, Kumamoto, Japan) was used to determine senescence-associated β-galactosidase activity according to the manual instructions.

CellTiter-Glo® 2.0 Cell Viability Assay (Promega) was used to determine the number of viable cells after senolysis.

### Isolation of primary myoblasts

Primary myoblasts were isolated from the skeletal muscles of C57BL/6J male mice [22]. All methods were carried out in accordance with the Guidelines for Proper Conduct of Animal Experiments by the Science Council of Japan. The experimental protocol used in this study was reviewed and approved by the experimental animal committee of the Kyoto Prefectural University of Medicine (Permit No. M2020-1). The study was carried out in compliance with the ARRIVE guidelines. Skeletal muscle tissues of the hindlimbs were digested with collagenase II (Sigma-Aldrich Japan, Tokyo, Japan). Dissociated cells were suspended in Ham’s F-10 medium (InvitrogenTM) containing 20% EquaFETAL (Atlas Biologicals) and 2 ng/mL of human recombinant basic fibroblast growth factor (bFGF) (Nacalai Tesque, Kyoto, Japan). They were seeded onto noncoated dishes and incubated at 37°C in 5% CO_2_ overnight to remove the fibroblasts. The next day, the supernatant was collected and centrifuged. The pellets were resuspended in Ham’s F-10 medium, which contains 20% EqualFETAL and 2 ng/mL of bFGF, and cultured on collagen-coated dishes until 70%–80% confluent.

### Statistics

The quantitative data are presented as the means ± SE. Data were analyzed using the Tukey–Kramer test, Dunnett′s test, or a t-test. p < 0.05 was considered statistically significant.

## Results

### Replicative senescence suppresses the myogenic differentiation of C2C12 cells

**Fig 1:**
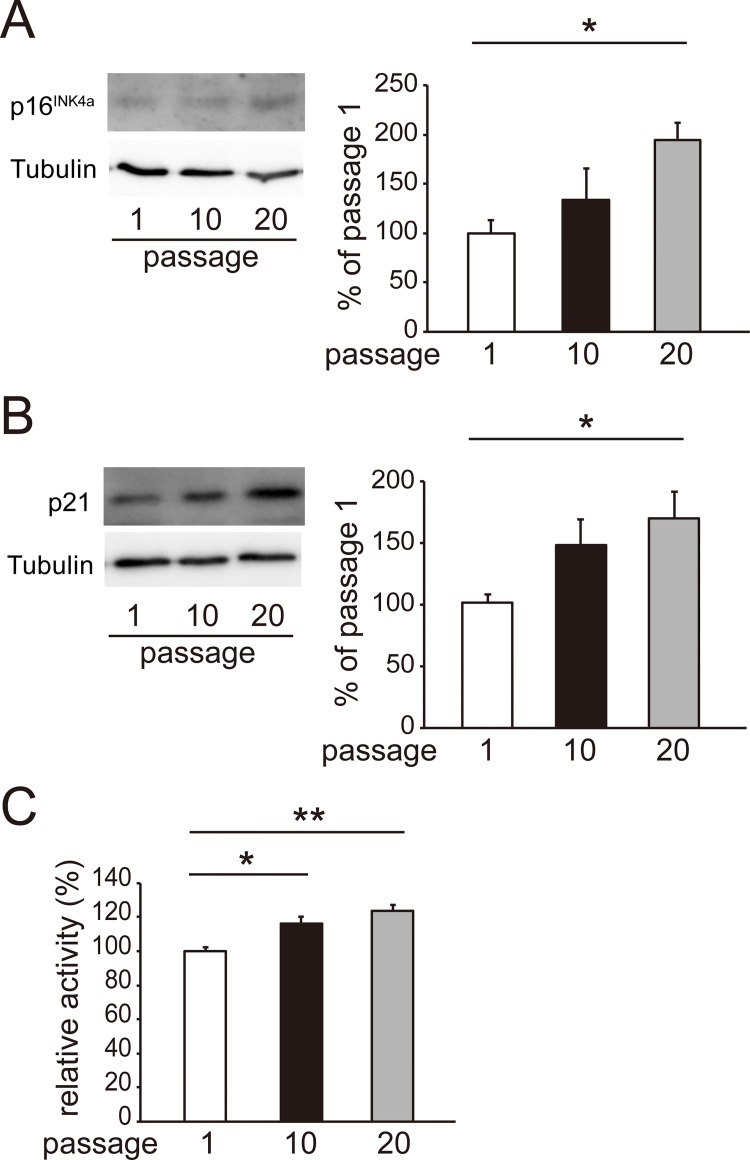
Induction of replicative senescence by high passage number in C2C12 cells. Panels A and B show representative western blots of p16^INK4a^ and p21 as senescence markers and tubulin as an internal control, respectively. Cells were subjected to passage once (passage 1), 10 times (passage 10), or 20 times (passage 20). The band density of p16^INK4a^ or p21 was normalized with that of tubulin. The normalized density was compared with the average of the normalized density in passage 1. Data are presented as means ± SE (n = 4 dishes). *P <0.05 (Dunnett′s test). Panel C shows the relative β-galactosidase activity. Data are presented as means ± SE (n = 6 wells). *P < 0.05, **P < 0.01 (Tukey–Kramer test).

**Fig 2:**
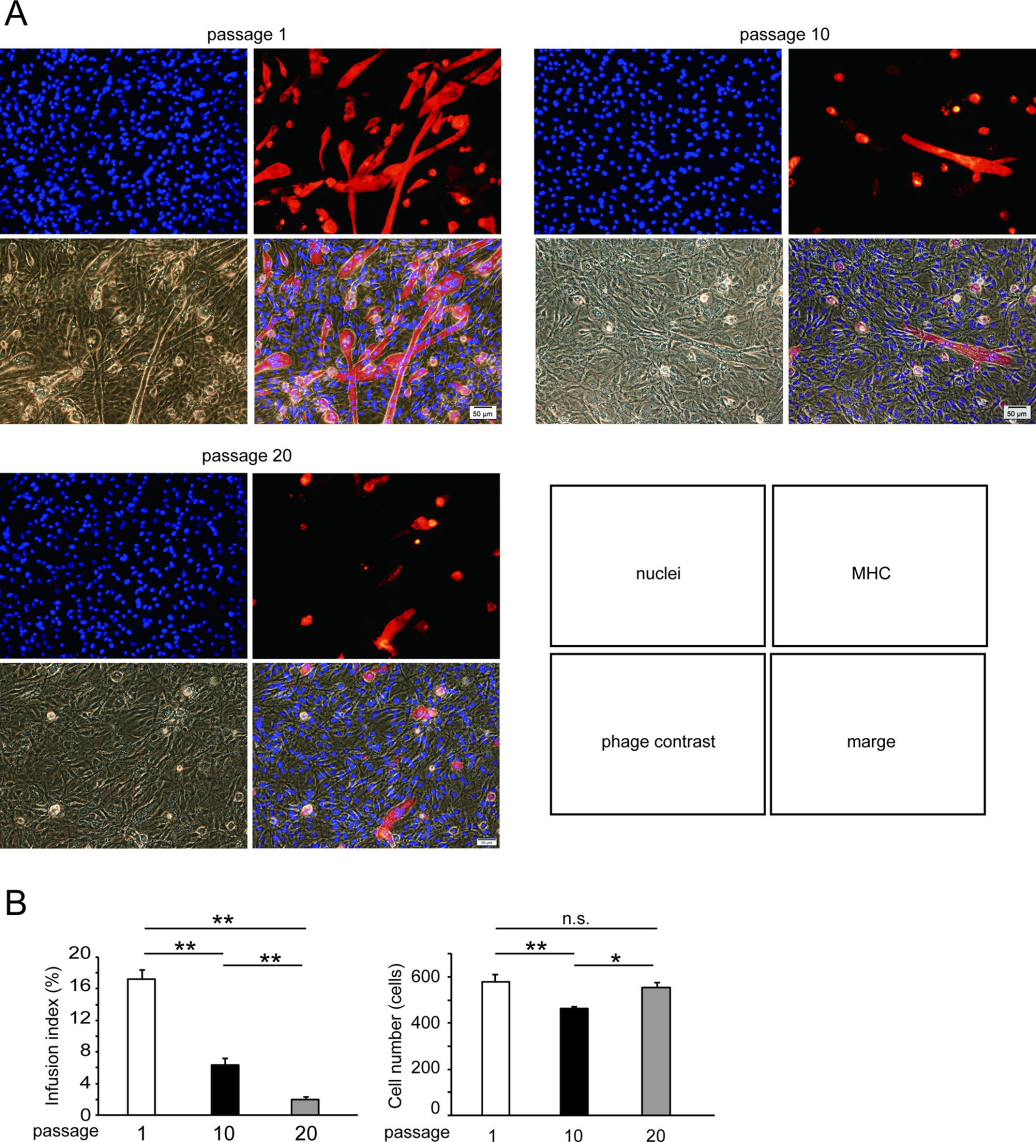
Effect of replicative senescence on myogenic differentiation of C2C12 cells. Panel A shows representative images of C2C12 cells after culture with differentiation medium for 7 days. Myogenic differentiation was induced in cells subjected to passage once (passage 1), 10 times (passage 10), or 20 times (passage 20). Red fluorescence of myosin heavy chain (MHC), blue fluorescence of nuclei, and phage contrast images were merged. Panel B shows the infusion index and the number of cells after myogenic differentiation for 7 days. Data are presented as means ± SE (n = 8 wells). *P < 0.05, **P < 0.01; n.s., not significant (Tukey–Kramer test).

**Fig 3:**
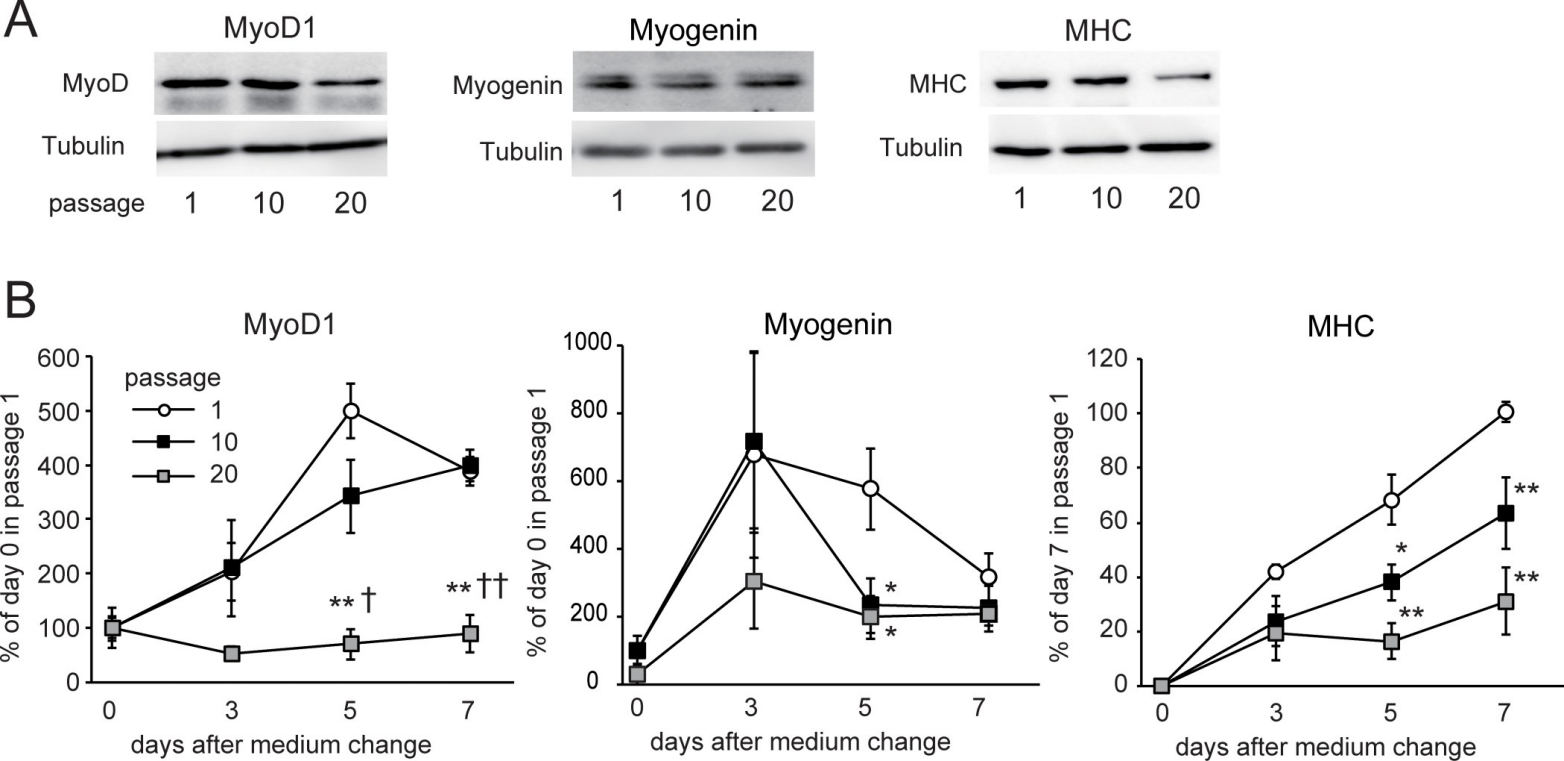
Effect of replicative senescence on myogenic marker expression during myogenic differentiation of C2C12 cells. Panel A shows representative Western blots of MyoD1, Myogenin, myosin heavy chain (MHC), and tubulin (as an internal control) in C2C12 cells after myogenic differentiation. Cells were collected 7 days after differentiation for MyoD and MHC or 5 days after differentiation for myogenin. Panel B shows the time course of MyoD1, myogenin, and MHC protein expression after changing the medium for myogenic differentiation. The band density of MyoD, myogenin, or MHC was normalized with that of tubulin. The normalized density was compared with the average of the normalized density in passage 1 just before myogenic differentiation (day 0) in the MyoD and myogenin expressions. Because MHC expression was undetectable on day 0, the normalized density was compared with the average of the normalized density in passage 1, 7 days after myogenic differentiation. Data are presented as means ± SE (n = 4 dishes). *P < 0.05, **P < 0.01 vs. passage 1 at each time point; †P < 0.05, ††P < 0.01 vs. passage 10 at each time point (Tukey–Kramer test).

To determine the effect of replicative senescence on the proliferation and differentiation of C2C12 cells, we repeated cell passage 10 or 20 times and induced myogenic differentiation by culturing the cells with DM for 7 days. The senescence markers p16^INK4a^ (Fig 1A) and p21 (Fig 1B) were induced by the 10th passage. Senescence-associated β-galactosidase was also activated by the 10th passage (Fig 1C). The infusion index decreased by 63% by passage 10 and by 88% by passage 20 compared with the control (passage 1) (Fig 2A and 2B). The cell numbers decreased by passage 10 but returned to those of the control level (passage 1) by passage 20. The expression of the MyoD1 and myogenin proteins, markers for the early phase of myogenic differentiation, and MHC, a marker for myotubes, was induced by DM incubation in passage 1 (Fig 3) However, the induction of MyoD1 and myogenin was decreased by passage 20 (Fig 3). The induction of MHC was suppressed in a stepwise manner by passages 10 and 20 (Fig 3). These results indicated that replicative senescence suppressed myogenic differentiation and that the effect was likely to expand with developing differentiation.

### Replicative senescence suppresses miR-1 and miR-133a expression

**Fig 4:**
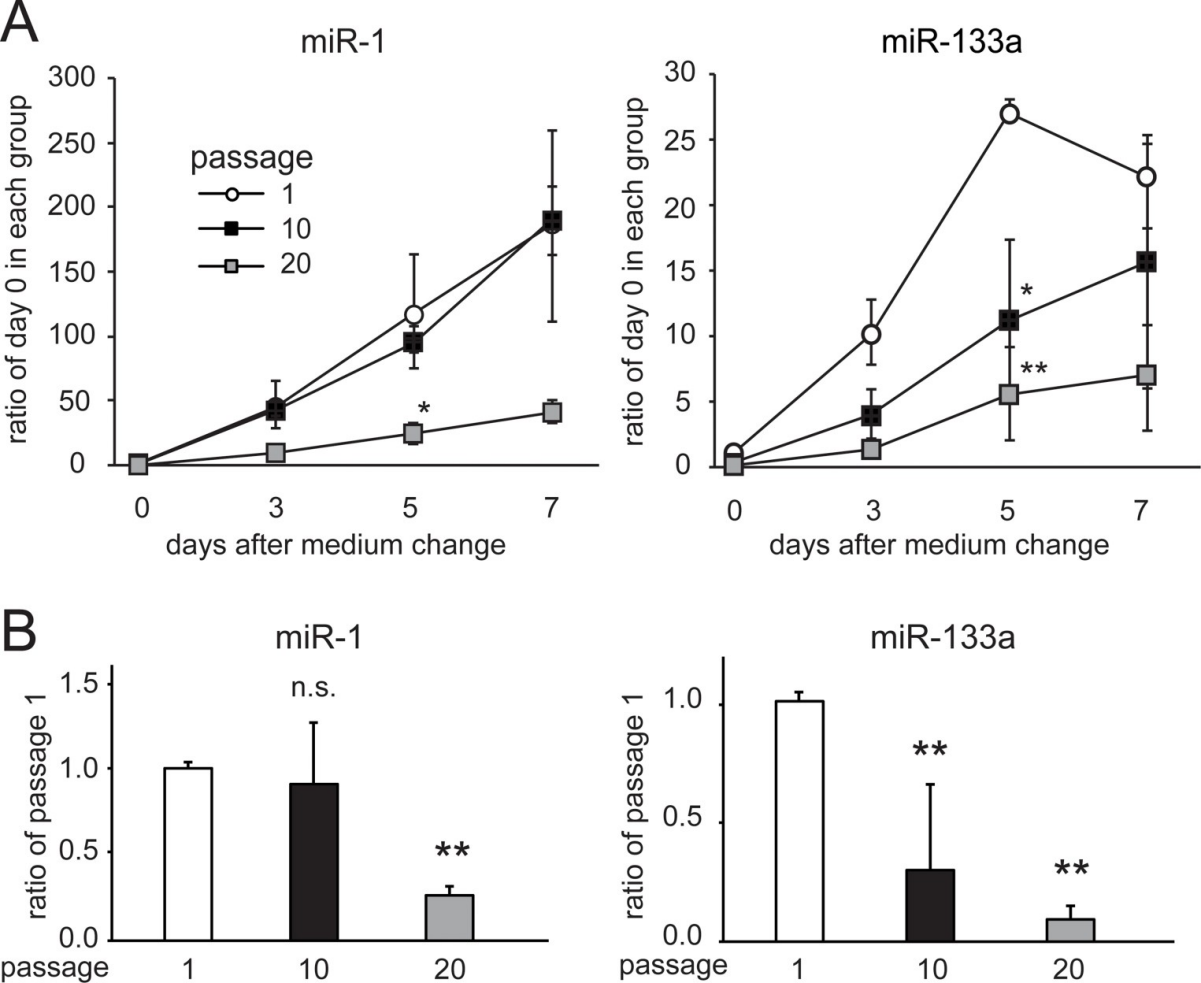
Effect of replicative senescence on miR-1 and miR-133a expression. Panel A shows the time course of miR-1 and miR-133a levels after changing the medium for myogenic differentiation. Myogenic differentiation was induced in cells subjected to passage once (passage 1), 10 times (passage 10), or 20 times (passage 20). Data are presented as means ± SE (n = 6 dishes). *P < 0.05, **P < 0.01 vs. passage 1 at each time point (Tukey–Kramer test). Panel B shows the relative levels of miR-1 and miR-133a in cells just before changing the medium for myogenic differentiation (day 0 in Fig 4A). Data are presented as means ± SE (n = 6 dishes). **P < 0.01 vs. passage 1 (Dunnett’s test).

Expression of either miR-1 or miR-133a gradually increased during culture with DM (Fig 4A). Expression of another muscle-specific miRNA cluster, miR-206/miR-133b, also increased during culture with DM (S1 Fig). The levels of miR-1 and miR-133a increased by 186-fold and 22-fold, respectively, 7 days after cell culture in DM (Fig 4A), while those of miR-206 and miR-133b increased by 15-fold and 11-fold, respectively (S1 Fig). The effects of replicative senescence on the expression levels of miR-1/miR-133a were examined. The induction of miR-1 expression was suppressed in cells at passage 20, but not in cells at passage 10. The induction of miR-133a was suppressed in cells at passage 10 and further in passage 20. These suppressive effects in replicative senescent cells had already appeared before myogenic differentiation was induced (Fig 4B).

### Overexpression of miRNAs ameliorates the differentiation capacity of senescent C2C12 cells

**Fig 5:**
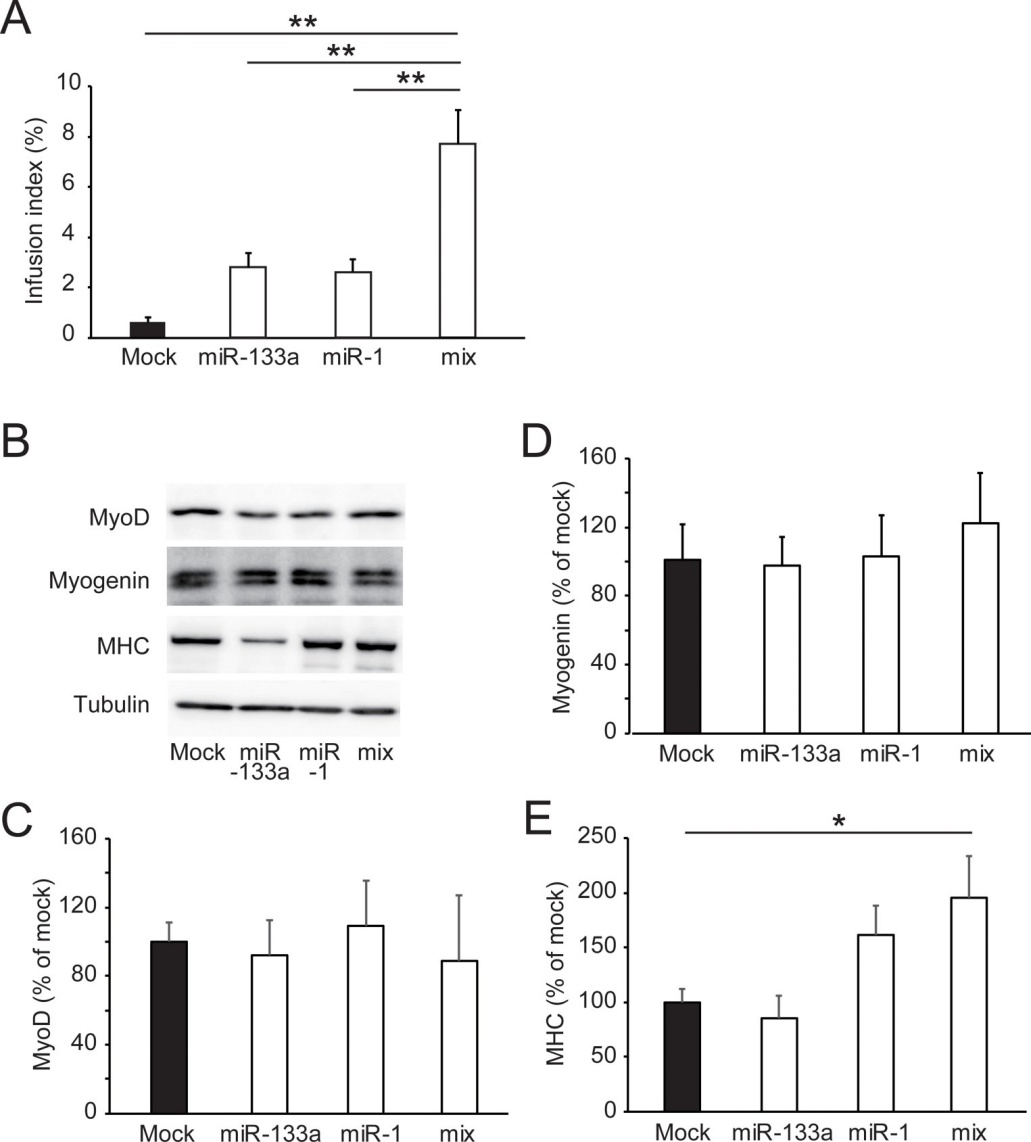
Effect of miRNA mimic transfection on myogenic differentiation in replicative senescent C2C12 cells. Panel A shows the infusion index after 7 days of differentiation in cells transfected with miR-133a, miR-1, or both. Mock cells were transfected with a negative control miRNA. Data are presented as means ± SE (n = 10 dishes). **P < 0.01 (Tukey–Kramer test). Panels B–E show representative blots of MyoD1, myogenin, MHC, and tubulin (B), and quantitative data (C–E) in cells after differentiation for 7 days. The band density of MyoD1, myogenin, or myosin heavy chain (MHC) was normalized with that of tubulin. The normalized density was compared with the average of the normalized density in the mock group. Data are presented as means ± SE (n = 5 dishes). *P < 0.05 (Tukey–Kramer test).

To determine whether the upregulation of miR-1 or/and miR-133a ameliorates the deterioration of myotube formation in senescent cells, miR-1 mimic, miR-133a mimic, or both were transfected into C2C12 cells with high passage numbers (passage 10) and the fusion index was determined. Because cells at the 20th passage could not survive lipofectamine treatment for miRNA transfection, the experiments on the 10th passage cells which were tolerant to transfection were carried out. MiR-1 and/or miR-133a were overexpressed in the transfected cells (S2 Fig). Transfection with miR-133a or miR-1 increased the infusion index compared with the negative control miRNA (Fig 5A). Co-transfection with miR-133a and miR-1 further improved the infusion index. Although there was no difference in MyoD1 and myogenin expression between mock cells and muscle-specific miRNA-transfected cells (Fig 5B–5D), the cells transfected with miR-1 or cells co-transfected with miR-133a and miR-1 exhibited increased MHC levels, whereas cells transfected with miR-133a did not express different MHC levels (Fig 5B and 5E). These results showed that the overexpression of both miR-1 and miR-133a was required to ameliorate the deterioration of myotube formation in senescent cells.

### Senolysis by glutaminase inhibitor increases miR-133a expression

**Fig 6:**
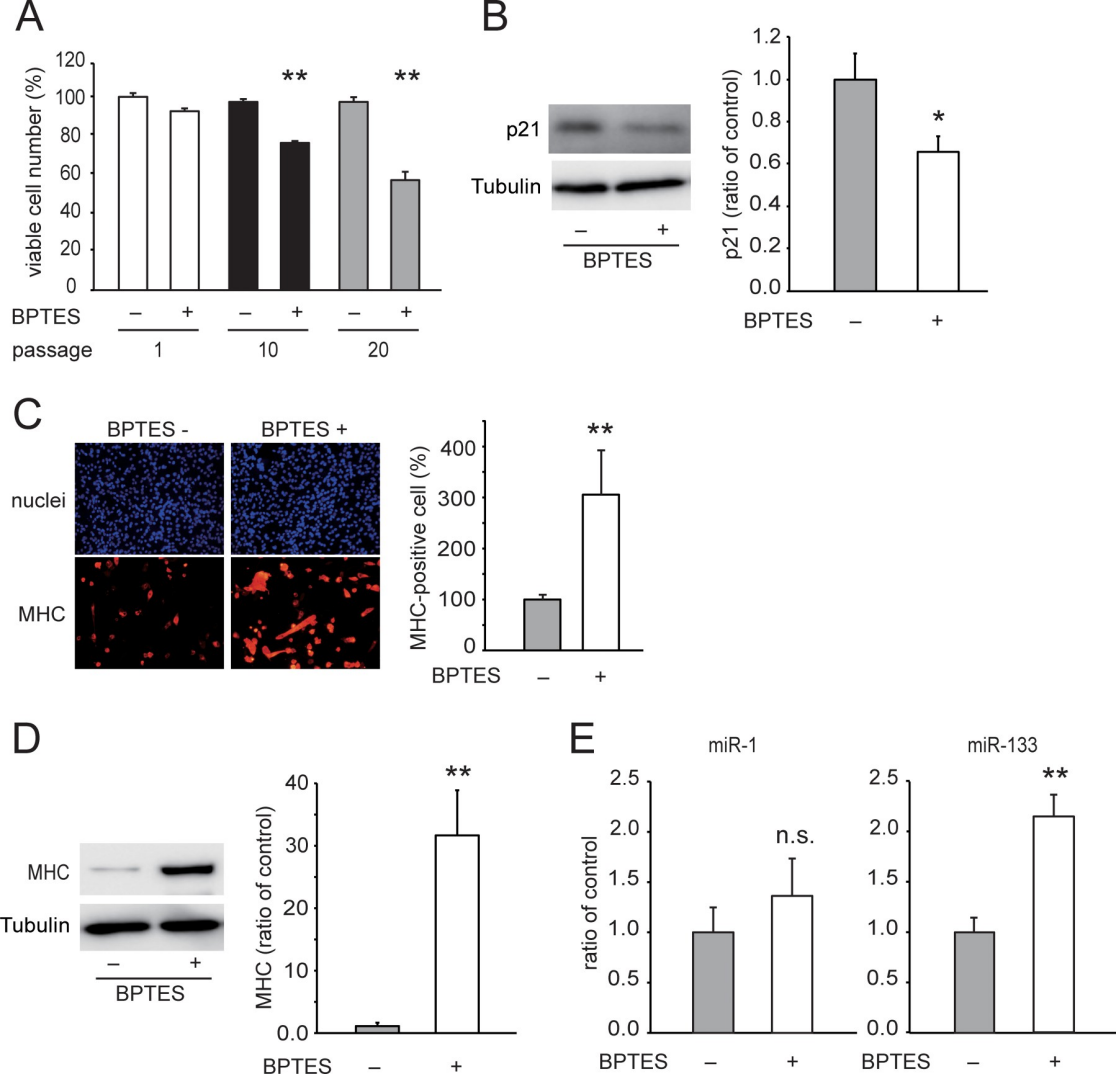
Effect of senolysis on the differentiation capacity of replicative senescent C2C12 cells. Panel A shows the relative cell number after treatment with BPTES or 1% DMSO for 48 h following repeating passage once (passage 1), 10 times (passage 10), or 20 times (passage 20). Data are presented as means ± SE (n = 8 wells). **P < 0.01 vs. 1% DMSO at each passage number (Tukey–Kramer test). Panel B shows representative blots of p21 and tubulin, and quantitative data normalized with the tubulin in cells surviving BPTES treatment following repeated passage 20 times. Data are presented as means ± SE (n = 4 dishes). *P < 0.05 (t-test). Panel C shows representative myosin heavy chain (MHC) immunostaining images and the number of MHC-positive cells. Cells that survived BPTES treatment following repeated passage 20 times were subjected to differentiation for 7 days and were stained with DAPI (blue fluorescence) and an antibody against MHC (red fluorescence). Data are presented as means ± SE (n = 7 dishes). **P < 0.01 (t-test). Panel D shows representative MHC and tubulin blots and quantitative data normalized with tubulin in cells surviving BPTES treatment following repeated passage 20 times. Data are presented as means ± SE (n = 4 dishes). **P < 0.01 (t-test). Panel E shows miR-1 and miR-133a levels in cells surviving BPTES treatment following repeated passage 20 times. Data are presented as means ± SE (n = 4 dishes). **P < 0.01 (t-test).

To determine whether the expression of miR-1 and miR-133a decreased as cellular senescence accelerated in C2C12 cells, senescent cells were removed by glutaminolysis inhibition and miRNA expression levels were measured in the surviving cells. Treatment with the glutaminase 1 inhibitor, BPTES [bis-2-(5-phenyl-acetamido-1,3,4-thiadiazol-2-yl) ethyl sulfide] [23], for 2 days removed 24% of the cells at passage 10 and 42% of the cells at passage 20, whereas it had no effect on the cells at passage 1 (Fig 6A). Cells that survived senolysis decreased p21 expression to 60% and increased differentiation capacity by 3-fold (Fig 6C) and MHC expression by 30-fold (Fig 6D) compared with untreated cells at passage 20. In surviving cells, the expression of miR-1 was slightly higher than that in untreated cells, whereas miR-133a levels increased two-fold (Fig 6E). These results indicated that cellular senescence decreased miR-1 and miR-133a expressions as well as differentiation capacity.

### Expression of miR-1 and miR-133a in myoblasts decreases in aged mice

**Fig 7:**
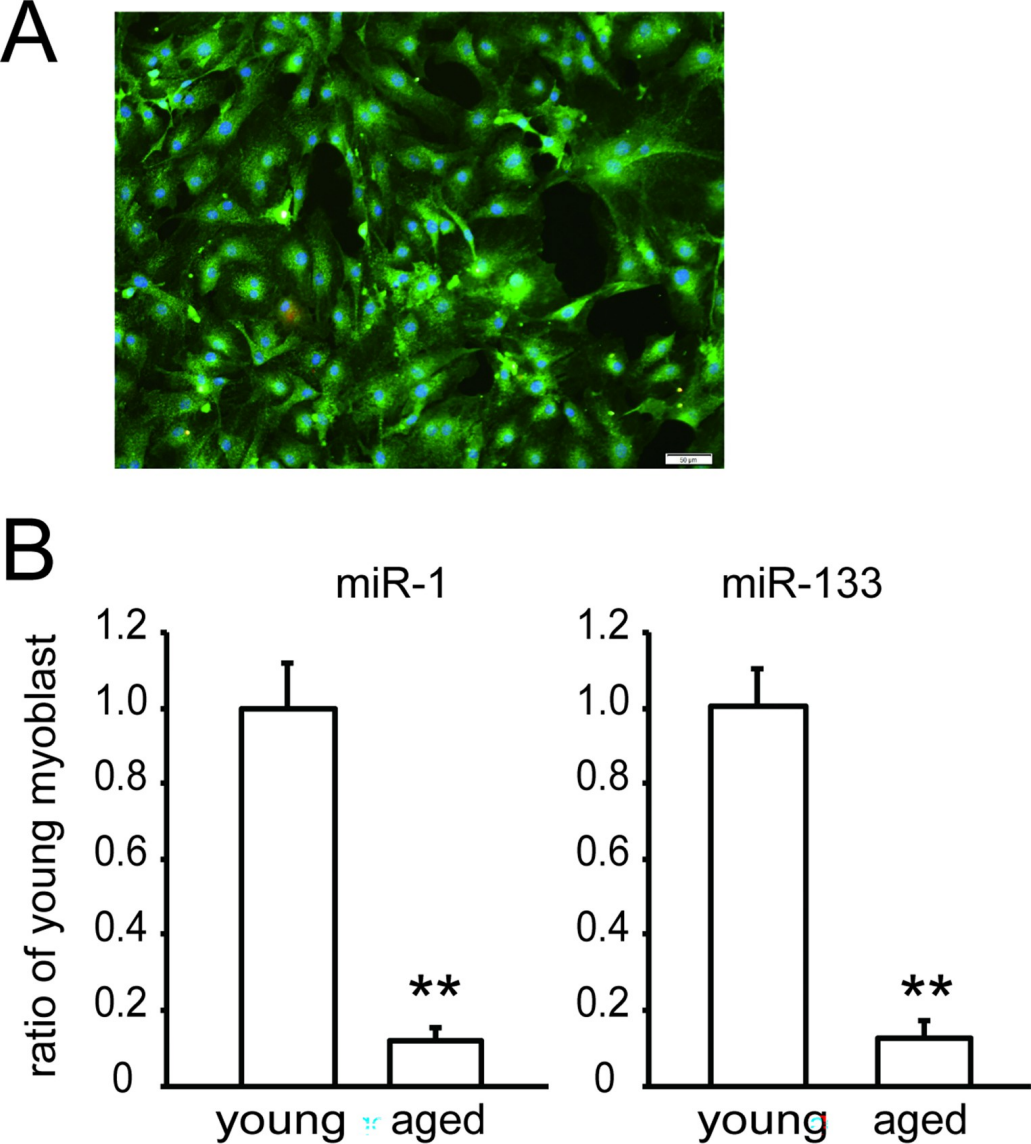
Comparison of miRNA levels in isolated myoblasts between young and aged mice. Panel A shows a representative immunocytochemical image of the isolated myoblast fraction. Green fluorescence of Pax 7, red fluorescence of myosin heavy chain (MHC), and blue fluorescence of nuclei images were merged. Panel B shows miR-1 and miR-133a levels in myoblasts isolated from young and aged mice. Data are presented as means ± SE (n = 4). **P <0.01 (t-test).

Myoblasts were isolated from young mice (3 months) and aged mice (20 months). The isolated myoblast fraction did not contain MHC-positive muscle cells (Fig 7A). The miR-1 and miR-133a levels in aged mice were only one tenth of those in young mice (Fig 7B) in the isolated myoblast fraction.

## Discussion

In this study, we showed for the first time that muscle-specific miRNA (miR-1 and miR-133a) expression in myoblasts is downregulated with replicative senescence using C2C12 myoblasts with a high passage number. These miRNAs in myoblasts from aged mice also decreased.

There have been several studies demonstrating the myogenic differentiation capacity of C2C12 cells declining at high passage number. Repeated passage up to 30 times reduced the length and area of myotubes formed from C2C12 cells [24]. Late passage up to 40 times impaired myocyte fusion and suppressed the expression of myogenic regulatory factors including MyoD1 [25]. In the present study, the infusion index began to decrease after the 10th passage (Fig 2), when replicative senescence was induced (Fig 1). Induction of MyoD1 and myogenin, early markers of myogenic differentiation, ceased by the 20th passage [25], whereas that of MHC was gradually suppressed after the 10th passage (Fig 3). These results suggest that myogenic differentiation becomes more senescent-sensitive with progression.

MiR-1 and miR-133a are organized as a bicistronic cluster and are thus transcribed together. Although miR-133a and miR-1 expressions were largely induced during myogenic differentiation, they decreased after the 10th and 20th passages, respectively. MiR-1 promotes myogenesis by inhibiting the expression of HDAC4, which represses the essential muscle-related transcriptional factors, MEF2C and MyoD1 [13]. At the same time, MyoD1 up-regulates miR-1 [26, 27]. Therefore, it is likely that both the induction of miR-1 and MyoD1 failed at the same passage number [25]. In contrast, the induction of miR-133a was suppressed after the 10th passage, when replicative senescence was induced and myogenic differentiation capacity deteriorated. In addition, the ameliorating effect of senolysis on miRNA expression was larger in miR-133a compared with miR-1 (Fig 6). Thus, it appeared that miR-133a was more sensitive to cellular senescence compared to miR-1. Expression of another muscle-specific miRNA cluster, miR-206/133b, also induced during myogenic differentiation, but the induction was less compared to that of the miR-1/133a cluster. In the miR-206/miR-133b knock-out mice, the deletion of the miR-206/133 cluster does not affect skeletal muscle regeneration in the Duchenne muscular dystrophy model mice [16]. The study suggested that the miR-206/133b cluster is likely to be dispensable for skeletal muscle regeneration because of the overlapping functions of the related miR-1/133a clusters [16]. The miR-1/133a cluster may be more dominant in myogenic differentiation.

Overexpression of either miR-133a or miR-1 ameliorated cell fusion in senescent C2C12 cells (Fig 5). Co-transfection with miR-133a and miR-1 further increased the fusion index. Misexpression of either miR-133a or miR-1 resulted in somite development defects in *Xenopus laevis* in vivo [15]. These findings suggest that the correct temporal expression and amount of both miR-133a and miR-1 are required for myogenic regeneration. In contrast, overexpression of miRNA-133a increased the infusion index without the induction of MHC expression (Fig 5). Tetraplex-binding porphyrin-treated C2C12 cells blocked MyoD1 transcription and ceased MHC induction but formed myotubes during myogenic differentiation [28]. MyoD1 induction was not repressed in cells at the 10th passage, and the transfection of miR-133a or miR-1 did not affect MyoD1 expression.

Sarcopenia is a condition characterized by the progressive and generalized loss of muscle mass, strength, and function [29, 30]. The prevalence of sarcopenia is related to skeletal muscle loss with aging [31]. Although the pathogenesis of sarcopenia remains unclear, sarcopenia is likely to be closely related to MuSC dysfunction [32]. MuSCs from 24-month-old mice exhibited a loss in migration speed, which is a key component of skeletal muscle regeneration [33]. The pool size and proliferation potential of MuSCs declined with age in mice [34]. In contrast, in satellite cell-depleted Pax7^CreER^-DTA mice, lifelong satellite cell reduction did not affect age-related myofiber atrophy and weakness, whereas it exacerbated the age-related excessive accumulation of extracellular matrix (ECM) in hind limb muscles [35]. Satellite cell reduction increased muscle fibroblast content, resulting in ECM accumulation [36]. Mice with deleted Trbp, an HIV TAR RNA-binding protein, exhibited reduced expression of miR-1 and miR-133a, impaired muscle regeneration and enhanced fibrosis in response to muscle injury [37]. Injection of miR-133a-overexpressed cardiac progenitor cells markedly improved cardiac function in a rat myocardial infarction model by reducing fibrosis and hypertrophy [38]. The role of muscle-specific miRNAs in myoblasts, including not only maintaining myogenic regeneration, but also protecting muscle fibrosis warrant further study.

## Supporting information

S1 Fig Time course of miR-206 (panel A) and miR-133b (panel B) levels after changing the medium for myogenic differentiation. Myogenic differentiation was induced in cells subjected to passage once (passage 1). Data are presented as means ± SE (n = 4 dishes).
(TIF)

S2 Fig The miR-1 (panel A) and miR-133a (panel B) expression levels in replicative senescent C2C12 cells transfected with miRNA mimic. Cells subjected to passage 10 times were transfected with miR-1, miR-133a, or both and collected before the induction of differentiation for miRNA quantitation. Mock cells were transfected with a negative control miRNA. Data are presented as means ± SE (n = 4 dishes). **P < 0.01 vs. mock (Dunnett′s test).
(TIF)

S1 Raw images Raw images of western blots for Figs 1, 3, 5 and 6. (PDF)
