# Supplementary figures and images for: Decrease in the expression of muscle-specific miRNAs, miR-133a and miR-1, in myoblasts with replicative senescence
Source: PLoS One. 2023 Jan 17;18(1):e0280527. doi: 10.1371/journal.pone.0280527 (PMC9844915; doi:10.1371/journal.pone.0280527)

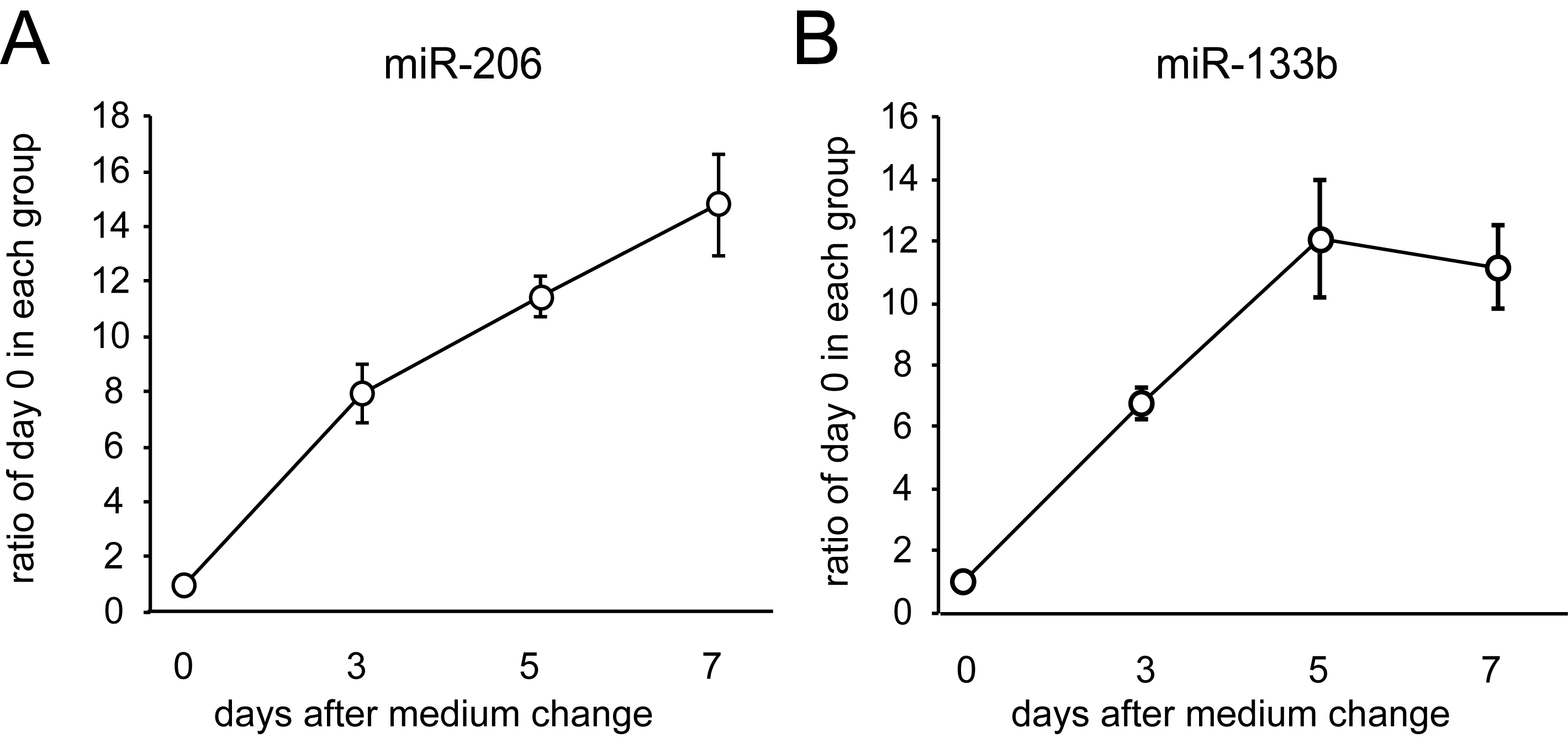

Supplement: S1 Fig — Time course of miR-206 (panel A) and miR-133b (panel B) levels after changing the medium for myogenic differentiation. Myogenic differentiation was induced in cells subjected to passage once (passage 1). Data are presented as means ± SE (n = 4 dishes). (TIF) [file pone.0280527.s001.tif]

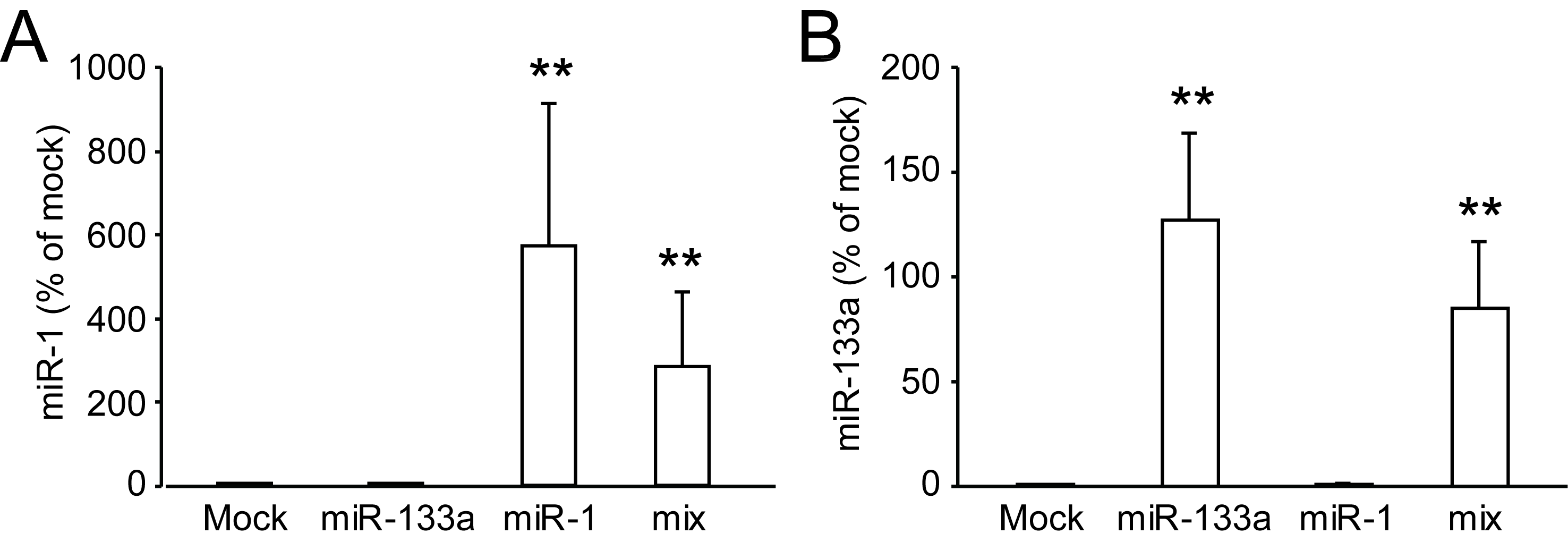

Supplement: S2 Fig — The miR-1 (panel A) and miR-133a (panel B) expression levels in replicative senescent C2C12 cells transfected with miRNA mimic. Cells subjected to passage 10 times were transfected with miR-1, miR-133a, or both and collected before the induction of differentiation for miRNA quantitation. Mock cells were transfected with a negative control miRNA. Data are presented as means ± SE (n = 4 dishes). **P < 0.01 vs. mock (Dunnett′s test). (TIF) [file pone.0280527.s002.tif]
