# Supplementary material for: Decrease in the expression of muscle-specific miRNAs, miR-133a and miR-1, in myoblasts with replicative senescence
Source: PLoS One. 2023 Jan 17;18(1):e0280527. doi: 10.1371/journal.pone.0280527 (PMC9844915; doi:10.1371/journal.pone.0280527)
Supplement: S1 Raw images — (PDF) [file pone.0280527.s003.pdf]

Fig 1A p16INK4a

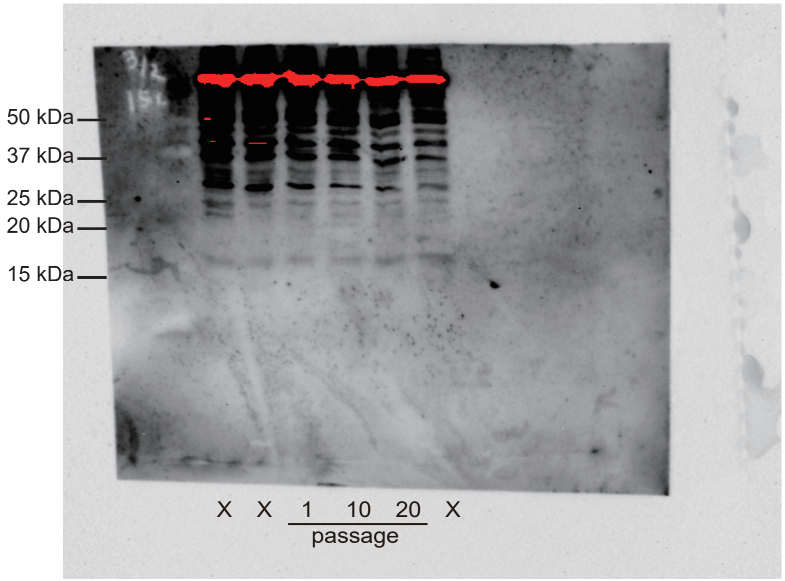

Fig 1A tubulin

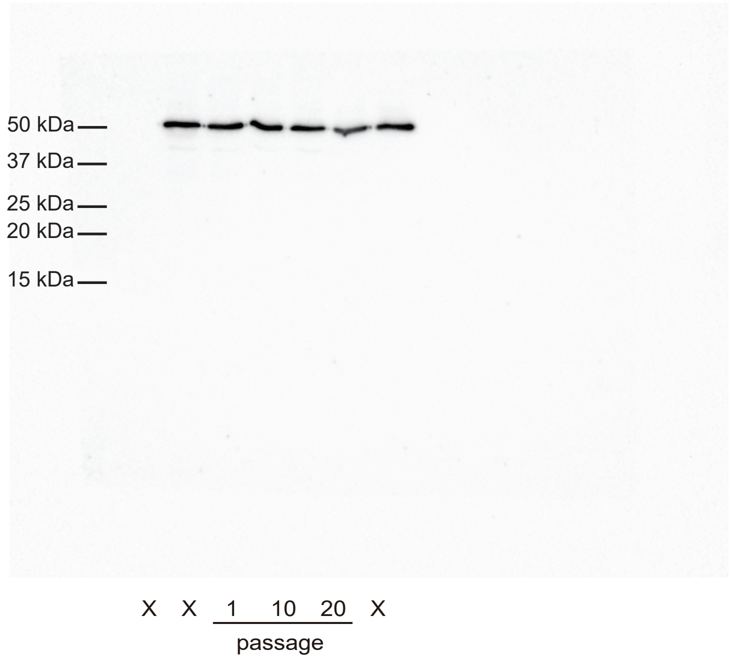

Fig 1B p21

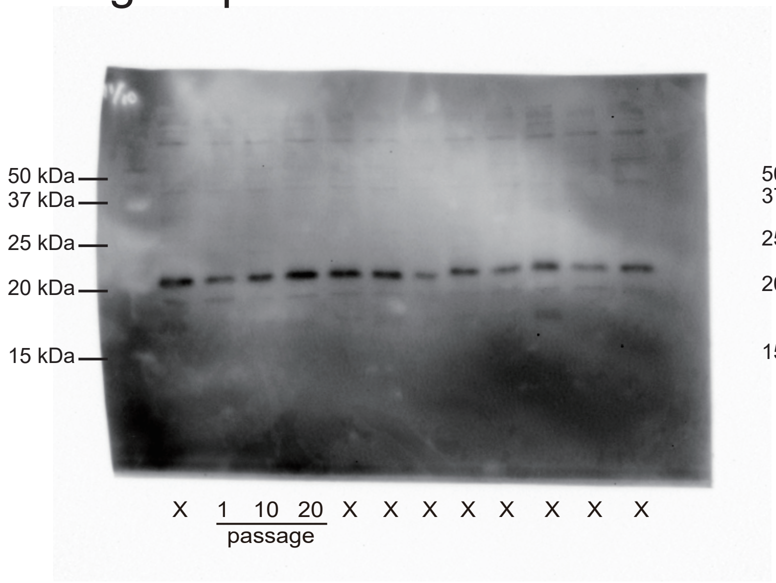

Fig 1B tubulin

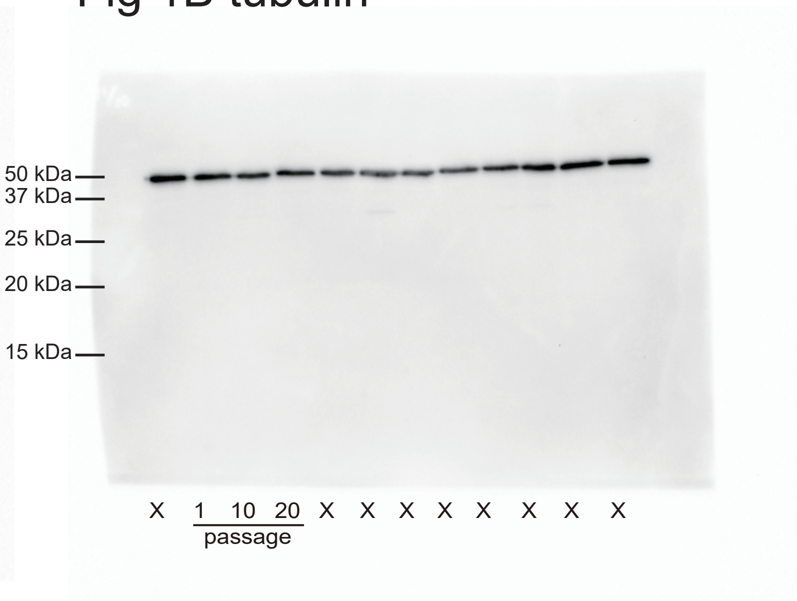

S3_raw_images

Fig 3A MyoD

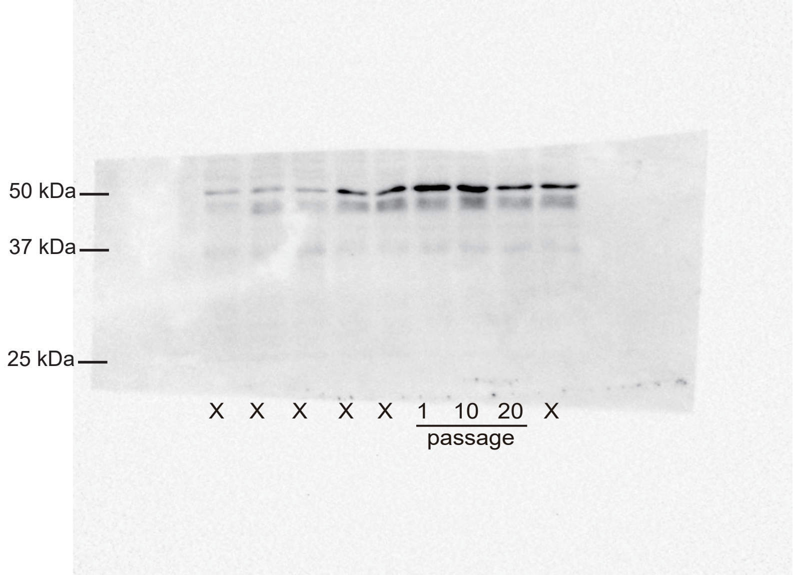

Fig 3A tubulin (for MyoD)

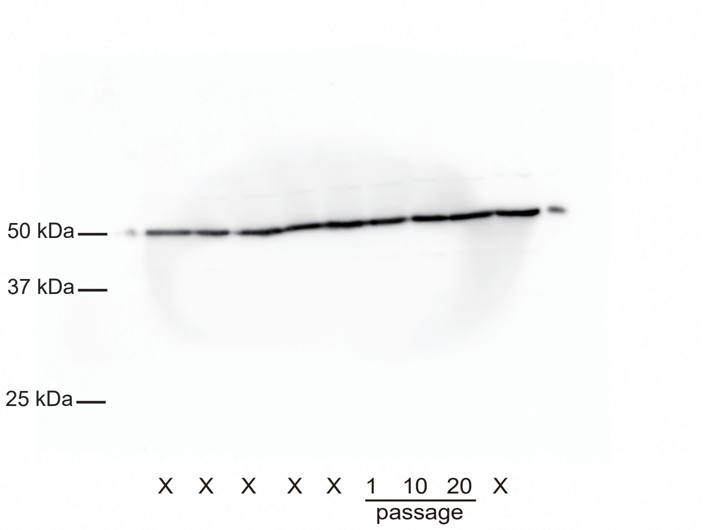

Fig 3A Myogenin

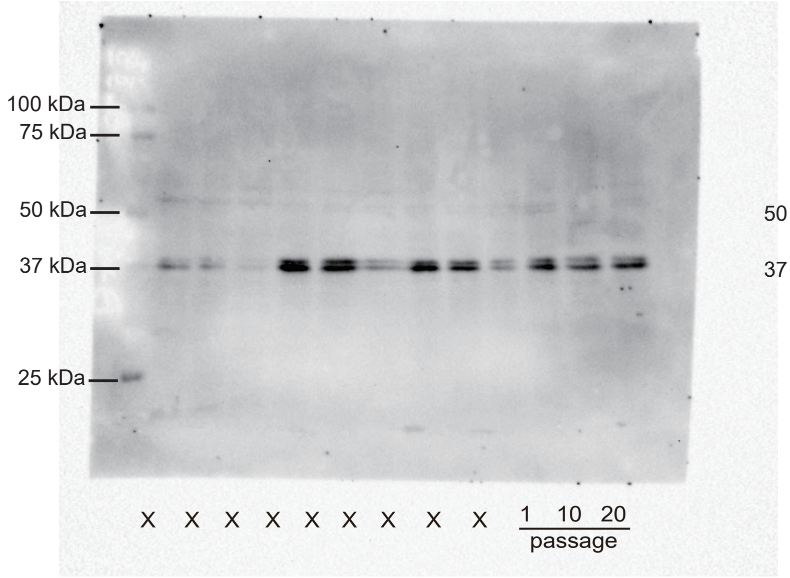

Fig 3A tubulin (for myogenin)

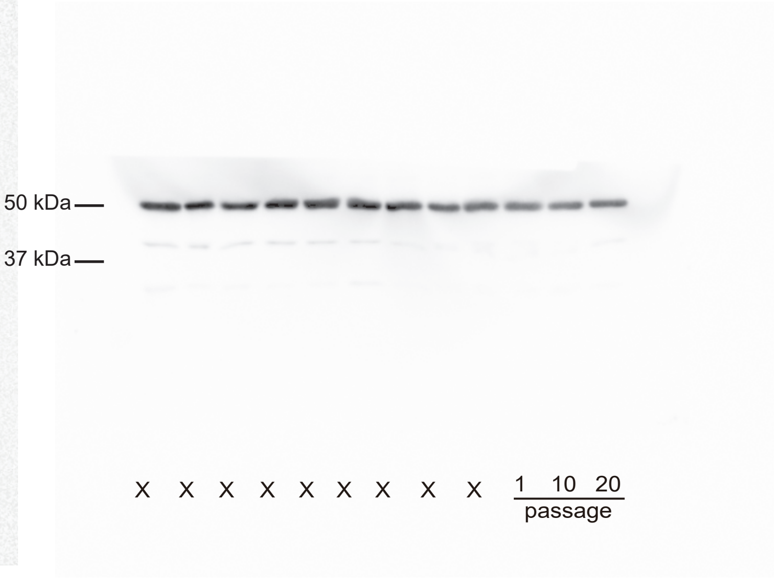

Fig 3A MHC

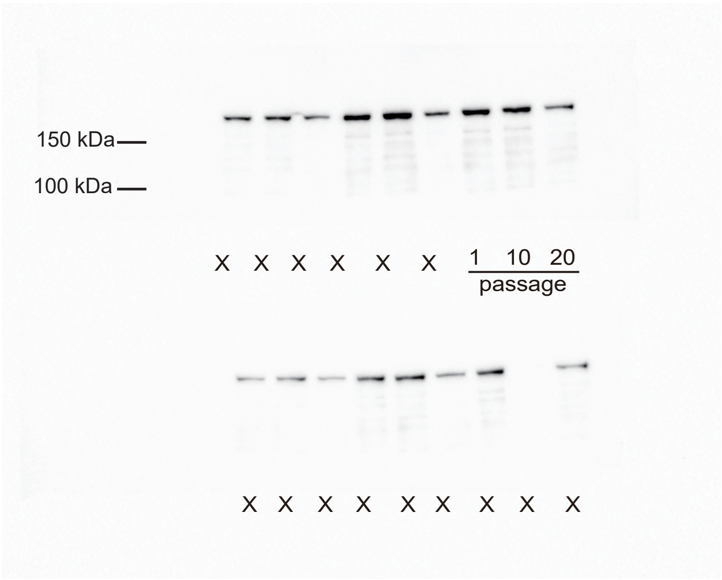

Two membranes were imaged simultaneously

Fig 3A tubulin (for MHC)

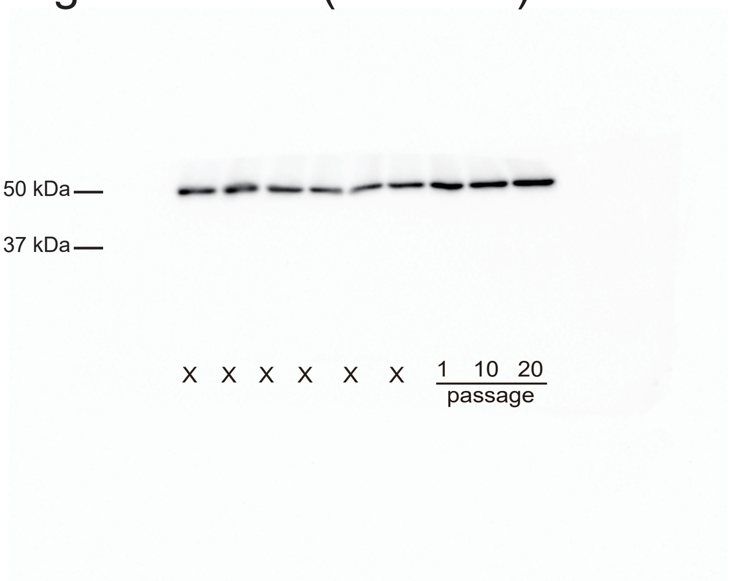

Fig 5B MyoD

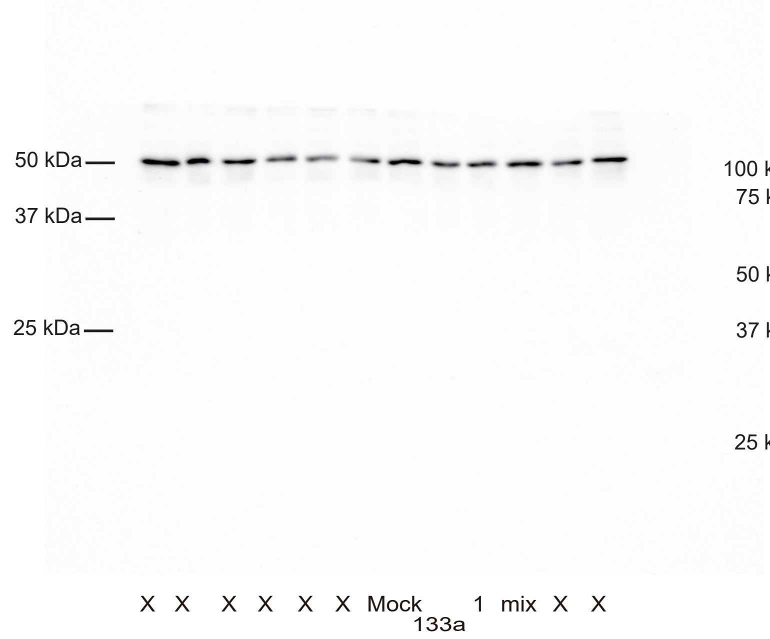

Fig 5B Myogenin

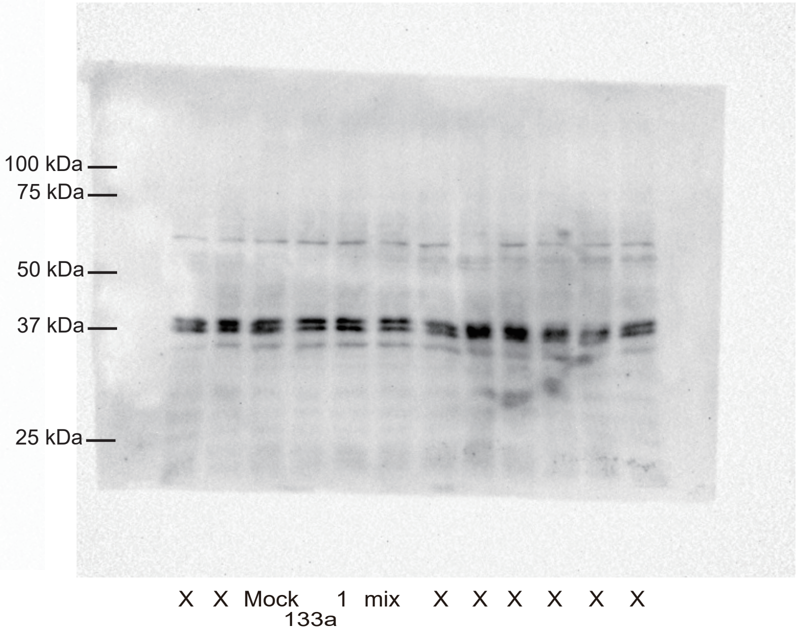

Fig 5B MHC

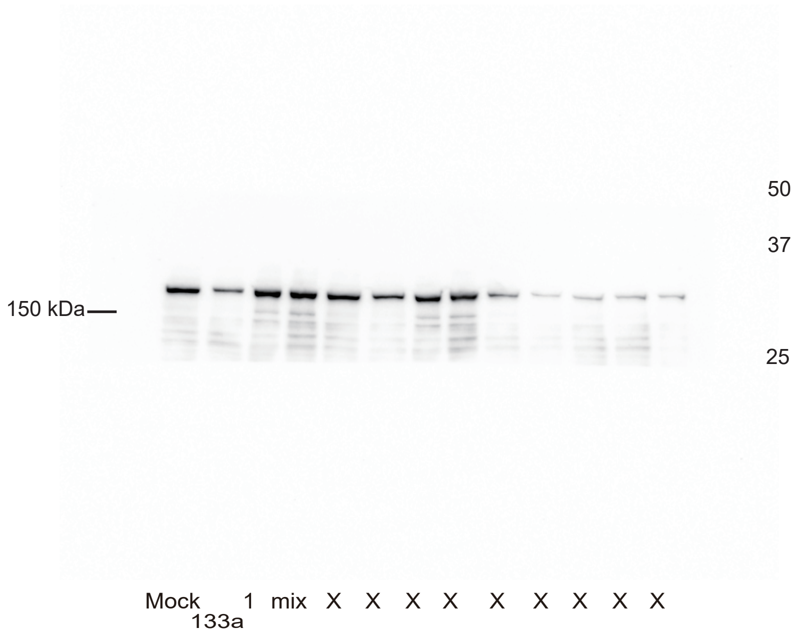

Fig 5B tubulin (for MHC)

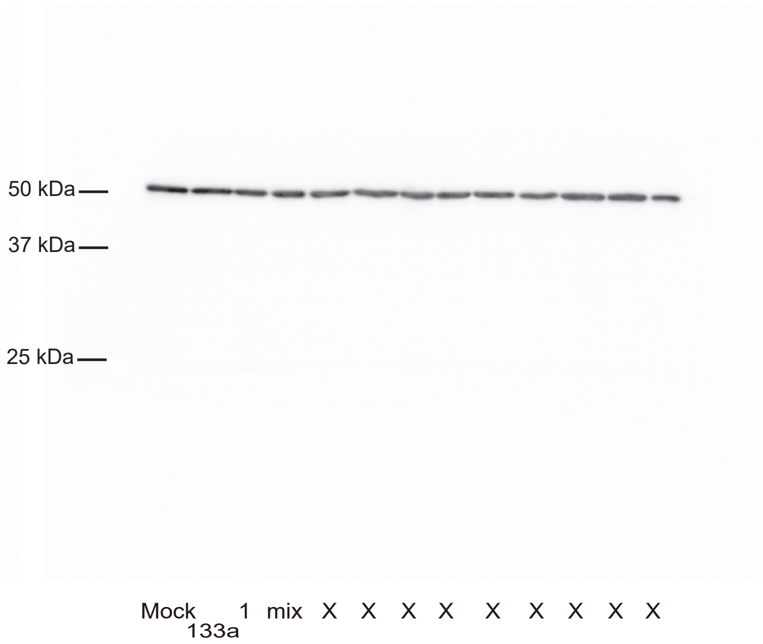

S3_raw_images

Fig 6B p21

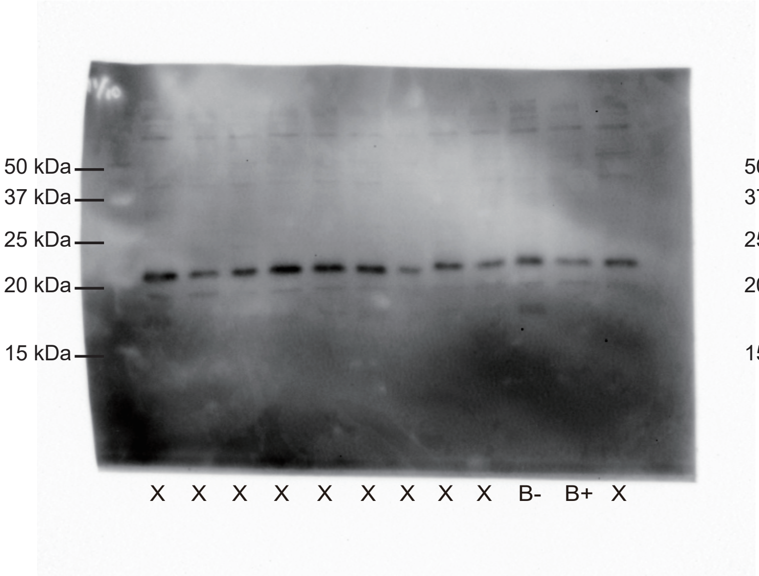

Fig 6B tubulin

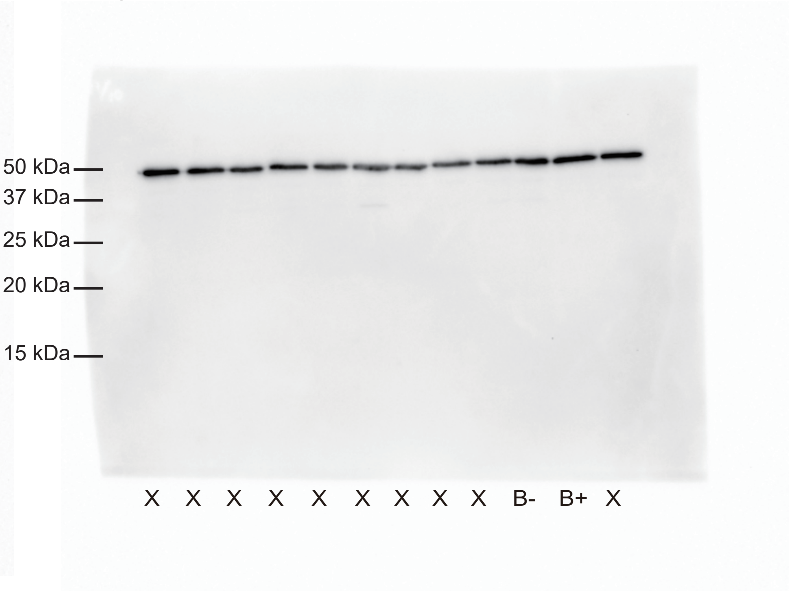

Fig 6D MHC

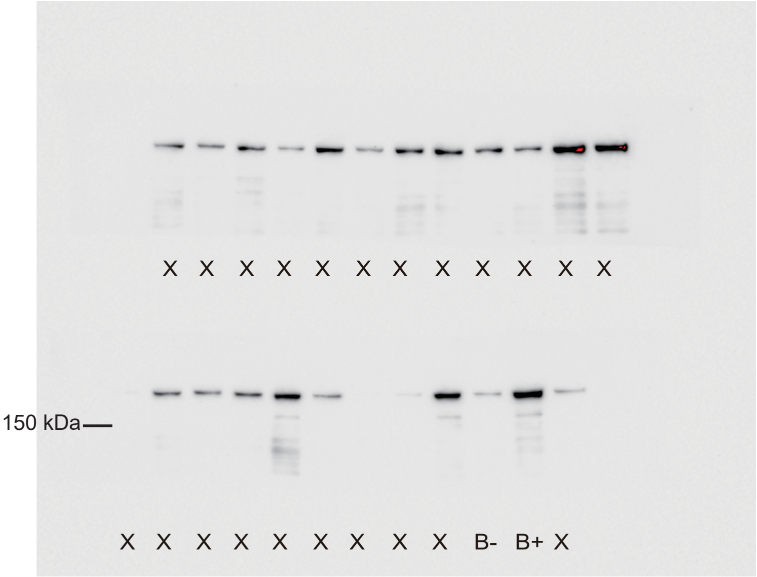

Two membranes were imaged simultaneously

Fig 6D tubulin

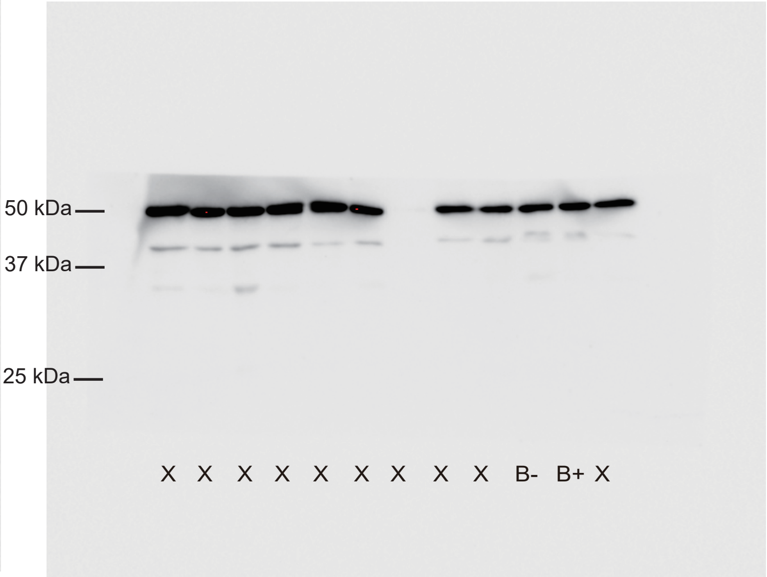

S3_raw_images
